# Dissolved organic carbon contribution to oxygen respiration in the central Red Sea

**DOI:** 10.1038/s41598-019-40753-w

**Published:** 2019-03-18

**Authors:** Maria Ll. Calleja, Najwa Al-Otaibi, Xosé Anxelu G. Morán

**Affiliations:** King Abdullah University of Science and Technology (KAUST), Division of Biological and Environmental Sciences and Engineering (BESE), Red Sea Research Center (RSRC), Thuwal, 23955-6900 Saudi Arabia

## Abstract

In oligotrophic waters, dissolved organic carbon (DOC) is mostly produced in the surface layers by phytoplankton and remineralized by heterotrophic prokaryotes throughout the water column. DOC surface excess is subducted and exported to deeper layers where a semi-labile fraction is further processed contributing to oxygen consumption. How this cycling of DOC occurs in the Red Sea, one of the warmest oligotrophic marine basins, is virtually unknown. We examined DOC vertical and seasonal variability in a mesopelagic station (ca. 700 m depth) of the central Red Sea performing monthly profile samplings over a two-year period. Together with DOC vertical and seasonal distribution we evaluated the interaction with heterotrophic prokaryotes and contribution to oxygen respiration. DOC values ranged from 41.4 to 95.4 µmol C L^−1^, with concentrations in the epipelagic (70.0 ± 7.5 µmol C L^−1^) 40% higher on average than in the mesopelagic (50.7 ± 4.1 µmol C L^−1^). Subduction of seasonally accumulated semi-labile DOC was estimated to be responsible for ∼20% of the oxygen consumption mostly occurring at the low epipelagic-upper mesopelagic boundary layer. Variability in mesopelagic waters was higher than expected (ca. 20 µmol C L^−1^) evidencing a more active realm than previously thought, with consequences for carbon sequestration.

## Introduction

The importance of the interactions between dissolved organic matter (DOM) and planktonic microbes in marine biogeochemical cycles is well recognized. With an estimate of 662 Pg C^1^, dissolved organic carbon (DOC) is the largest pool of reduced carbon in the biosphere and comparable in size to the atmospheric CO_2_ pool. In the oligotrophic ocean, organic carbon is mostly produced by phytoplankton in the surface layers. Heterotrophic prokaryotes (HP) are in turn responsible for most of DOC remineralization throughout the water column, although photochemical oxidation could also play a role in highly UV-irradiated surface waters by making some organic molecules more susceptible to microbial remineralization^2,3^. The net conversion of CO_2_ into organic material through photosynthesis in the upper layers and ulterior export and processing in the ocean’s interior, known as the biological carbon pump, represents a net removal and long-term storage of atmospheric CO_2_^1^, playing a key role in the regulation of global climate^4,5^. The efficiency of carbon transport and storage depends on the fraction of the system’s net production removed from the surface waters via export mechanisms. It is therefore essential to quantify the magnitude of carbon export from the surface waters to the deep ocean, not only to understand the current Earth’s carbon cycle but to be able to better assess the impact of continuously increasing atmospheric CO_2_ levels.

Well-recognized export processes from the surface to the oceans interior are the sinking of particulate organic carbon (POC) and the transport of DOC by turbulent mixing. Together with the production of long-lasting DOC through microbial processes, known as the microbial carbon pump^6^, and the enzymatic and mechanical solubilisation of POC throughout the water column^7^, all contribute to carbon sequestration. However, active transport of both POC and DOC by vertically migrating animals is another known but not yet quantified carbon transfer mechanism to the ocean interior^8,9^ with consequences for the microbial community^10,11^. The relative contributions of POC and DOC transport to carbon sequestration are still unclear. DOC is estimated to account for ~20% of global export, but it can vary largely (more than threefold) across oceanographic regions^12^, accounting for as much as half of the total export in oligotrophic subtropical gyres^13^. As these stratified and nutrient-depleted regions may expand with increased warming^14^, the role of DOC in the global carbon transfer to the deep sea may be intensified. However, our understanding of DOC dynamics and cycling in oceanic regions experiencing increase stratification is still scarce and needs to be addressed. This study focuses on DOC dynamics, export and cycling in the central Red Sea, a tropical oligotrophic semi-enclosed marine basin undergoing faster warming rates (0.17 ± 0.07 °C decade^−1^) than the global ocean (0.11 °C decade^−1^)^15^ and experiencing intense stratification in the upper layers. Altogether, this makes the Red Sea a marine ecosystem that can serve as a model to study other tropical marine basins threatened by rapid surface warming.

To understand DOC dynamics it is necessary to somehow decipher the heterogeneous mix of molecules with turnover times ranging from minutes to millennia that comprise the bulk DOC pool. In order to describe its complexity, three major fractions are commonly distinguished according to turnover or life time: labile (minutes to days), semi-labile (months to years) and refractory (centuries to millennia)^16^. The lability continuum of DOC pool in the oceans is defined by the capability of heterotrophic prokaryotes to breakdown its compounds and use them for respiration and/or self-biomass production. The labile DOC fraction is freshly produced mostly in the photic layer as a result of photosynthesis and food web processes^17^, and consumed very quickly; therefore, it does not accumulate and is usually studied in incubation experiments^18,19^. The semi-labile DOC fraction escapes rapid microbial degradation and it can seasonally accumulate in the surface waters in conditions of high stratification. This accumulated fraction can be transported downward by vertical mixing and convective overturn and plays the most important role from the carbon transport perspective^1,16^. The subducted semi-labile DOC can be then remineralized when exposed to subsurface nutrients and heterotrophic prokaryote community with the right enzymatic activities^20,21^. The refractory fraction, the nature of which is still under debate^6,22^, is the most ubiquitous one, present in all depths. Refractory DOC has a mean age of 3700–6000 years according to ^14^C records^23^, making it the biggest and longer lasting fraction stored in the deep ocean. However, this is the average age of a non-homogenous pool that comprises from relatively young compounds to truly ancient carbon sources (>20000 years old)^24^. The DOC lability and potential for degradation, transport and accumulation depends on its nature and chemical composition, not easy to disentangle due to its high heterogeneity. However, different molecular, spectroscopic and separation techniques are in continuous development in order to shed light on its chemical structure^25^. Among others, the study of the optical fraction of DOM that can emit fluorescence after absorbing light in the UV and visible range, called fluorescent DOM (FDOM) and ubiquitously present in the oceans, has proved a valuable tool to provide information on the reactivity and composition of DOC in the oceans^26,27^. In this study we examine FDOM properties in order to diagnose the lability and degradation state of the bulk DOC pool.

Besides analyzing its chemical properties, the lability of subducted DOC can also be explored by evaluating how much of it is being used by heterotrophic prokaryotes through respiration or oxygen utilization. The relationships between changes in DOC concentration and apparent oxygen utilization (AOU) below the thermocline allow us to quantify the remineralization of DOC by microbial respiration and its relative contribution to total oxygen utilization. AOU integrates all respiratory processes within a water body since it is isolated from the atmosphere, therefore precluding oxygen exchanges with it, and it reflects the oxidation of the total biogenic carbon (POC and DOC)^28^. Relationships between DOC and AOU have been widely studied^29–33^. The contribution of DOC remineralization to changes in AOU appears to vary largely, with values ranging from <20% in equatorial regions to ~70% in subtropical gyres^13,30,34^. The large spatial variability observed across different oceanographic regions makes the contribution of DOC to respiratory processes still unclear. The higher the accumulation of semi-labile DOC in stratified oligotrophic waters^29^, the higher would be the net DOC transport downward that can potentially be respired by heterotrophic prokaryotes. Most of the studies examining the relationship of DOC and AOU mentioned above have used measurements restricted to one or two consecutive months of a particular year. However, DOC accumulation depends on the decoupling of microbial consumption from primary production in the euphotic layer, which is tightly coupled to seasonal changes^35^. Thus, we believe that to properly assess the contribution of DOC to AOU at a particular location, seasonal studies measuring DOC and AOU along the water column covering a full annual period would more realistically predict DOC contribution to respiration.

In this study we have simultaneously evaluated the vertical and temporal changes of DOC and AOU in a mesopelagic station (ca. 700 m depth) of the central Red Sea over a two-year period, and examined their relationships with the communities of heterotrophic prokaryotes inhabiting both the epipelagic and mesopelagic layers. We provide a detailed assessment of the seasonality of DOC, HP and AOU in a tropical open water stratified ecosystem off the Red Sea central coast.

## Methods

### Study site

The data were obtained from repeated samplings performed at a mesopelagic station in the central Red Sea, off King Abdullah Economic City (KAEC, 22.47 °N 39.03 °E) between November 2014 and May 2017, covering a two-year period. All samplings were performed around midday on board the KAUST *R.V. Thuwal* and KAUST *R.V. Explorer*.

### Hydrography data

Vertical profiles down to 700 meters depth (site seabed) of salinity (Sal, S.I.), water temperature (T, °C), dissolved oxygen (O_2_, mg L^−1^), and vivo fluorescence (Fluor, R.U.) were recorded continuously in a conductivity-temperature-depth (CTD) probe SeaBird 11plus V 5.2 system, with a pre-calibrated through Winkler titration polarographic membrane oxygen sensor SeaBird-43 and a Wet Labs ECO-AFL/FL fluorometer installed in the rosette sampler. Apparent Oxygen Utilization (AOU) was calculated as the difference between the oxygen saturation and the measured dissolved oxygen. Oxygen saturation represents the oxygen concentration at equilibrium with the atmosphere at the temperature and salinity observed and was calculated using the equation of Benson and Krause^36^. Potential density or Sigma-Theta (σ_θ_, Kg m^−3^) was calculated from *in situ* salinity, potential temperature, and pressure = 0, minus 1000 Kg m^−3^ ^37^. Potential density gradient was used to estimate the mixed layer depth (MLD). MLD was determined for each profile as the depth where a change in density higher than or equal to 0.05 Kg m^−3^ was first observed for a 5 m interval.

### Sample collection and analysis

Discrete water samples were collected from niskin bottles at 12–16 depths distributed throughout the whole water column for the analysis of chlorophyll a (Chl *a*), dissolved organic carbon, inorganic nutrients and heterotrophic prokaryote abundance.

### Chlorophyll a analysis

Samples for Chlorophyll *a* (Chl *a*) analysis were collected from 8 profiles at 5–6 depths distributed above 100 meters and including the depth of maximum fluorescence or deep Chlorophyll maximum (DCM) identified by the CTD attached fluorometer. 250 ml of water from each depth was collected from the niskin bottles and kept at 4 °C until filtration (performed within the next 2 hours of sample collection). Unfortunately Chl *a* samples from summer time were not collected. Chl *a* concentration was obtained after filtration of 200 ml water samples through 0.22 µm size filter (IsoporeTM Membrane Filters (RTTP)). Filters were collected and kept frozen (−80 °C) until further analysis. Pigments were then extracted in 90% acetone solution for 24 h in darkness at 4 °C and Chl *a* pigment concentration was measured with a Turner Trilogy fluorometer calibrated with pure Chl *a* standard^38^.

### DOC analysis

Samples for DOC analysis were passed through an online acid-cleaned polycarbonate filter cartridge, holding a pre-combusted (450 °C, 4.5 h) GF/F filter, attached directly to the Niskin bottle, and collected into acid cleaned and pre-combusted glass vials. Samples were acidified with H_3_PO_4_ until pH 1–2, and kept in the dark at 4 °C until analysis at the laboratory. A total of 296 DOC samples collected were analysed by high temperature catalytic oxidation (HTCO) in a Shimadzu TOC-L. All glass material used was previously acid cleaned and burned (450 °C, 4.5 h). Three to five replicate 100 µl of sample were injected into the combustion tube pre-heated to 680 °C. The resulting peak area was then integrated with Shimadzu chromatographic software. A five-point calibration curve of potassium hydrogen phthalate solution was performed daily to standardize the instrument response. All samples were systematically referenced against Consensus Reference material of deep-sea water (DSW, 42–45 μmol C L^−1^) and low carbon water (LCW, 1–2 μmol C L^−1^), provided by D. A. Hansell (Univ. of Miami), to monitor the ultimate accuracy of the measurements, allowing a resolution of 1.4 µmol L^−1^.

Changes in DOC concentration along the thermocline (∆DOC/∆z) were calculated and used as a proxy for vertical export. The decrease of DOC concentration per meter was calculated for each profile as the absolute slope of the DOC concentration change with depth (∆DOC/∆z) within the thermocline, and its absolute value expressed in µmol C L^−1^ m^−1^.

### FDOM analysis

Samples for FDOM analysis were collected from the same GF/F filtrate into acid-cleaned and pre-combusted 40 ml glass bottle. UV-VIS fluorescence spectroscopy was measured immediately after collection using a HORIBA Jobin Yvon AquaLog spectrofluorometer installed onboard, with a 1 cm path length quartz cuvette. Three dimensional fluorescence excitation emission matrices (EEMs) were recorded by scanning the excitation range between 240 and 600 nm and emission wavelength range of 250–600 nm, both at 3 nm increments and using an integration time of 8 seconds. To correct and calibrate the fluorescence spectra post-processing steps were followed according to Murphy *et al*.^39^. Briefly, Raman-normalized Milli-Q blanks were subtracted to remove the Raman scattering signal^40^. All fluorescence spectra were Raman area (RA) normalized by subtracting daily blanks that were performed using Ultra-Pure Milli-Q sealed water (Certified Reference, Starna Cells). To extract information of the fluorescent signal of obtained EEMs the fluorescent, humification and biological indices were evaluated. The fluorescence index (FI) was calculated as the fluorescence intensity ratio at 450 nm and 500 nm emission and 370 nm excitation^41^. The humification index (HIX) was obtained as the ratio of peak area under emission spectra between 435 and 480 300–345 nm at an excitation wavelength of 254 nm^42^. HIX index has been long used as an indicator of the humic substance content or the extent of humification^43^. However, its significance in the oligotrophic ocean is still unclear and novel interpretations have been recently put forward^44^, which will be further discussed. The biological index (BIX) was obtained as the ratio of emission at 380 and 430 nm at 310 nm of excitation wavelength^45^ and is an indicator of autotrophic productivity, with values >1 revealing recently produced DOM of autochthonous origin^46^.

### Inorganic nutrients

Samples for dissolved inorganic nutrients analysis were collected from the same GF/F filtrate into 15 ml acid cleaned Falcon tubes and stored frozen at −20 °C until analysis. Nitrate (NO_3_^−^) nitrite (NO_2_^−^) and phosphate (PO_4_^3−^) were analyzed by colorimetry on a Bruan and Luebbe® Autoanalyzer following the methods described at Hansen and Koroleff^47^ for the automated analysis in segmented flow. Limits of quantification were 0.2, 0.06 and 0.01 µmol L^−1^ for NO_3_^−^, NO_2_^−^ and PO_4_^3−^ respectively. All standards were prepared with a nutrient-free artificial seawater matrix in acid-washed glassware. DIN = [NO^−3^] + [NO^−2^] (note that NH_4_^+^ was not measured), and DIP = [PO_4_^3−^], in μmol N L^−1^ and μmol P L^−1^, respectively.

### Heterotrophic prokaryotes

Heterotrophic prokaryote samples were collected in sterile Falcon tubes from each niskin. Subsamples were then fixed with 1% paraformaldehyde + 0.05% glutaraldehyde (final concentration), kept for 10–15 min in the dark, deep-frozen in liquid nitrogen, and stored at −80 °C until further analysis. In the laboratory aliquots of 0.4 ml were stained with SYBR Green I (Molecular Probes) (1:10000 final dilution of initial stock) and run using a BD FACSCanto flow cytometer following the methodology described in more detail in Gasol and Morán^48^. 1.0 μm fluorescent latex beads (Molecular Probes, ref.) were added to each sample as internal standards used to calibrate the flow cytometric size signals. Samples were analyzed at low flow rates until the acquisition of 10000 events occurred and abundance was estimated after daily calibrating the flow rate. Flow cytometry files were further processed using the FCSExpress 5 software.

### Data treatment and statistical analysis

Contouring of the data, statistical analysis and graphs were performed using the JMP statistical package and software (SAS Institute Inc.). Correlations between the investigated variables were examined with parametric Pearson correlations. p-values below 0.05 are considered statistically significant. A linear regression (model II) was performed to infer the discussed equations. To study the seasonal variability data was grouped in northern meteorological seasons. To evaluate significant differences between seasons a one-way analysis of variance (ANOVA) followed by a Fisher’s Least Significant Differences post hoc test (posthoc Fisher LSD test) was performed using MATLAB_R2015b. To study vertical variability data was grouped in two main layers: the water layer above 200 m, or epipelagic (<200 m); and the water layer bellow 200 m, or mesopelagic (200–700 m). Supplementary Table 1 shows ANOVA results and posthoc seasonal significances of all variables for both the epipelagic and mesopelagic layers. Both main layers were further examined along different isopycnal layers (see detailed information below). Unless otherwise stated values reported within the text represent mean ± standard deviation.

## Results

### Hydrological features, Chl a fluorescence and AOU distribution

**Figure 1:**
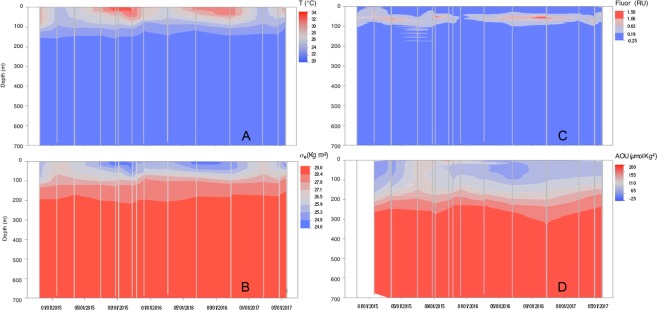
Contour plots of hydrological features, fluorescence and AOU. Dynamics of the vertical profiles of (**A**) temperature (°C), (**B**) density or Sigma-theta (σ_θ,_ Kg m^−3^),(**C**) fluorescence (R.U) and (**D**) apparent oxygen utilization (AOU, µmol Kg^−1^) during the two-year sampling. Vertical grey lines represent the casts performed.

Figure 1A illustrates the vertical and temporal distribution of temperature along the two-year studied period. Surface temperature varied from 24.6 °C (February 2017) to 32.7 °C (August 2015) and it decreased with depth to a uniform temperature of 21.6 °C from 200 m down to the sea floor. The permanent thermocline was found between 100 and 200 m while the seasonal one was observed at shallower depths, especially during early summer (20 m). The hot (>30 °C) surface water layer extended down to 50 m during the second half of the summer. Salinity also showed a seasonal pattern in the upper 200 m, with higher salinities during summer and fall (reaching values as high as 39.8 in October 2016) and minimum values (38.0) in winter. The seasonal halocline was located within the upper 50 m. Below 200 m salinity remained fairly constant at 40.6. The potential temperature versus salinity graph (Supplementary Fig. 1) shows a clear distinction between the thermohaline properties of the water masses present in the upper 100 meters during the different seasons, with high salinity (>39.2) and temperature (>30 °C) waters in summer and fall, and colder and fresher waters in winter. Potential water density or Sigma-Theta (σ_θ_, Kg m^−3^) varied from 24.10 to 28.80 (Fig. 1B). It showed a seasonal variability in the upper 200 m, with lower values during summer and fall and higher values during winter and spring (Supplementary Table 1). The MLD averaged 38 ± 16 m and also showed a clear seasonality, displaying deeper values, 47 ± 14 and 48 ± 12 m, during winter and fall respectively, and shallower values 33 ± 12 and 21 ± 2 m, during spring and summer respectively (Supplementary Fig. 2B).

Chlorophyll *a* fluorescence distribution (Fig. 1C), recorded on the CTD attached fluorometer, showed a persistent deep chlorophyll maximum (DCM). The DCM was deeper (between 45 and 75 m) and more intense during summer and fall 2016, than during winter (broader peak found at around 50 m) although the strongest DCM in 2015 occurred earlier in the year, during spring. Chlorophyll *a* fluorescence seasonality for the whole epipelagic layer is indicated in Supplementary Table 1. Total Chl *a* concentration of discrete water samples collected at the DCM depth varied between 0.28 and 0.43 µg L^−1^ while surface values ranged from 0.10 to 0.18 µg L^−1^.

AOU vertical and seasonal distribution is illustrated in Fig. 1D and Supplementary Table 1. Summer and fall displayed high levels of AOU in the upper 150 m (between 70 and 170 µmol Kg^−1^) compared to winter and spring, when values for the same layer were generally lower than 70 µmol Kg^−1^. Values close to zero were found during spring and winter-time in the upper 75 meters. Deeper in the water column AOU values up to 200 µmol Kg^−1^, indicative of highly oxygen under-saturated water, were observed from 300 m downwards.

### Inorganic nutrients

DIN and DIP exhibited surface concentrations ranging from below detection limits to 1.21 and 0.17 µmol L^−1^, respectively. Their values increased with depth along the thermocline to mean values of 19.6 ± 3.8 and 0.94 ± 0.29 µmol L^−1^ in mesopelagic waters. No seasonality was observed for DIN or DIP in surface waters, which remained depleted in inorganic N and P year round, or in mesopelagic waters. DIN:DIP ratios in the epipelagic (11 ± 10), however, were seasonally variable, with significantly higher values in summer (20 ± 7) than in the rest of the seasons (10 ± 2) (Supplementary Table 1). Mesopelagic DIN:DIP ratios averaged 23 ± 7 and did not show any significant seasonal pattern (Supplementary Table 1).

### Heterotrophic prokaryotes distribution

**Figure 2:**
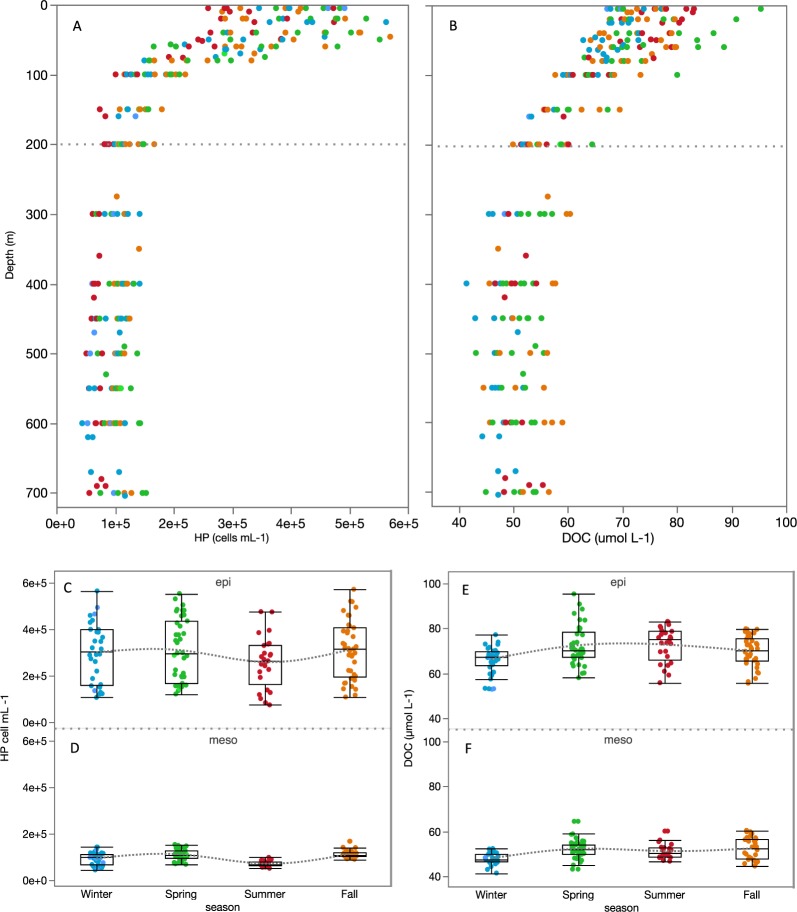
DOC and HP vertical and temporal variability. Vertical profiles of HP abundance (cells mL^−1^) (**A**) and DOC concentration (µmol C L^−1^) (**B**) for the whole dataset. Seasonal variability in the epipelagic and mesopelagic layers of HP abundance (**C**,**D**) and DOC concentration (**E**,**F**). Seasons are indicated in different colors: Winter (blue), Spring (green), Summer (red) and Fall (orange).

Heterotrophic prokaryotes vertical distribution is illustrated in Fig. 2A. As expected, close to one order of magnitude higher abundances were found in the epipelagic (0.4 to 2.5 × 10^5^ cells ml^−1^) than in the mesopelagic layer (1.9 to 9.7 × 10^4^ cells ml^−1^). Seasonal changes were significant in the mesopelagic (Supplementary Table 1) and a similar pattern was observed in both the epipelagic and mesopelagic layers (although with much smaller variability in the mesopelagic layer), with highest abundances observed in spring and lowest in summer (Fig. 2C,D).

### DOC vertical and temporal variability

**Figure 3:**
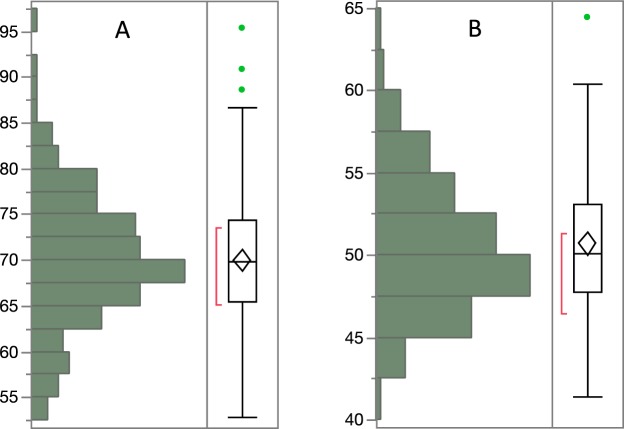
DOC distribution in the epipelagic and mesopelagic. Distribution of DOC concentration (µmol C L^−1^) in the epipelagic (**A**) and mesopelagic (**B**) layers. The horizontal line in the boxes represents the median, and the diamond sign represents the mean. The boxes extend from the 25% to the 75% quartile of the data distribution, and the vertical lines indicate the 5% and 95% quartiles. Circles outside the boxes represent individual data that falls below or above the 5% and 95% quartiles. The red line indicates the best fitting normal distribution for that data.

DOC values ranged from 41.4 to 95.4 µmol C L^−1^. As expected, DOC showed the highest concentrations in the epipelagic (Fig. 2B), with a mean value of 70.0 ± 7.5 µmol C L^−1^, followed by a marked decrease within the thermocline. A mean value of 50.7 ± 4.1 µmol C L^−1^ was found in the mesopelagic layer. Seasonal changes were significant and more evident for the epipelagic (Fig. 2E) than the mesopelagic layer (Fig. 2F) (Supplementary Table 1), with lower and less variable concentrations during the winter, and higher values during periods of stronger stratification (spring and summer). Overall, DOC concentrations in the epipelagic and mesopelagic layers (Fig. 3) showed similar ranges of variability, with respective coefficients of variation of 11% and 8%.

### FDOM variability

**Figure 4:**
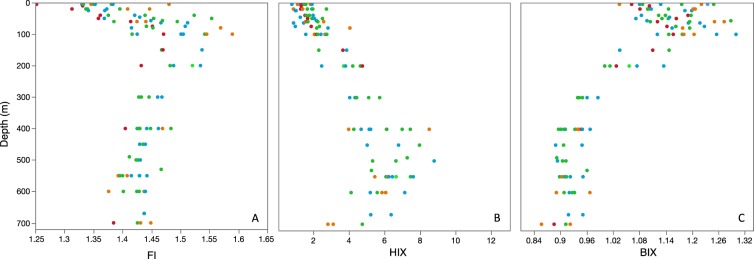
FDOM vertical variability. Vertical profiles of DOM optical indices: Fluorescent index (FI, **A**), humification index (HIX, **B**) and biological index (BIX, **C**). Seasons are indicated in different colors: Winter (blue), Spring (green), Summer (red) and Fall (orange).

With the exception of a fluorescence index (FI) value of 1.25, the FI ranged between 1.3 and 1.6 (Fig. 4A), notably smaller than values recently reported for the Mediterranean Sea^27^, suggesting that the existing FDOM was a mix of terrestrial-like (FI∼1.2) and marine microbial origin (FI∼1.8)^43^. FI was highly variable in the epipelagic, with lowest values at the surface and a gradual increase until around the DCM. FI slightly decreased again from 100 to 200 m and remained relatively constant (1.53 ± 1.38) in mesopelagic waters. The humification index (HIX), indicative of DOM of lower C/N ratios^43^, ranged between 0.8 and 8.9 (Fig. 4B), similar to values reported for the Mediterranean^27^. Smaller values were also observed in surface waters and increased almost linearly until ca. 400 m. Between 400 and 550 m DOM seemed to reach its highest degree of humification, and values gradually decreased again from 550 until the sea floor. The biological index (BIX), indicative of recent biological activity or fresh DOM, varied between 0.9 and 1.3 (Fig. 4C), slightly higher than values reported in the Mediterranean^27^. Higher values were found in the upper 100 m, decreasing gradually between 100 and 400 m. Values remained low although a subsequent decrease was found close to the sea floor. A weak seasonality was observed in the epipelagic for FI and BIX indices (Supplementary Table 1), with lower values observed in summer (1.4 ± 0.07 and 1.1 ± 0.04, respectively) and higher values in fall (1.5 ± 0.08 and 1.2 ± 0.07, respectively).

### DOC surface excess and vertical export

The vertical transport of DOC from the surface layer to the mesopelagic (defined as the introduction of surface accumulated DOC to depths >200 m) depends on the decrease of DOC concentration within the thermocline and the water renewal time or ventilation time. Assuming a constant ventilation time for the two-year sampling period (a recent study estimated a renewal time in the Red Sea to be of the order of a decade^49^), we have used changes in DOC concentration along the thermocline (∆DOC/∆z) as a proxy for vertical export. Higher DOC vertical export will occur at higher DOC concentration gradients. Pooling all profiles together, the ∆DOC/∆z averaged 0.143 ± 0.029 µmol C L^−1^ m^−1^, and it showed a clear seasonal pattern (Supplementary Fig. 2A) inversely related to the seasonality observed for the MLD (Supplementary Fig. 2B). Significantly higher slopes were observed during stratified summer conditions (0.183 ± 0.014 µmol C L^−1^ m^−1^, p < 0.05, posthoc Fisher LSD test) compared to winter (0.122 ± 0.016 µmol C L^−1^ m^−1^) (Supplementary Fig. 2A).

### Relationships between DOM (DOC and FDOM), inorganic nutrients, and AOU

With all data pooled, DOC significantly decreased with increasing AOU (r = −0.80, p < 0.0001, slope = 0.15). However, a DOC-AOU relationship for the entire water column is of limited value because it combines depth layers where it may differ. Therefore, an evaluation of the two properties along isopycnal layers was performed. To better understand processes occurring during DOC remineralization, the relationship between fluorescent indices (HIX and BIX), and dissolved inorganic nutrients (DIN, DIP) with AOU were similarly evaluated when DOC and AOU showed a significant correlation. Pearson correlations results of all variables in each isopycnal are shown in the Supplementary Table 2.

Five different isopycnal layers were evaluated according to the density distribution (σ_θ_) shown in Fig. 1B.

Isopycnal 1 was the shallowest density layer (σ_θ_ = 24–26.5 Kg m^−3^), mostly comprising the upper 60 m. Within this layer DOC tended to increase with increasing AOU, but the correlation was weak and not significant, probably due to processes other than heterotrophic respiration, such as primary production oxygen, exchange with the atmosphere, and DOC photo-oxidation, affecting changes in both DOC and dissolved oxygen concentrations in the surface waters.

Isopycnal 2 (σ_θ_ = 26.5–27.1 Kg m^−3^), mostly between 60 and 100 m, thus encompassing the DCM, and isopycnal 3 (σ_θ_ = 27.1–27.8 Kg m^−3^), a thin layer around 100 m, displayed no clear relationship between DOC and AOU, indicating that heterotrophic oxygen consumption was not yet enough to significantly deplete DOC concentrations within these depth intervals. Another possible explanation could be that DOC may simultaneously increase as a consequence of POC solubilization from marine snow aggregates^50^, thus obscuring the relationship between DOC consumption and AOU buildup.

**Figure 5:**
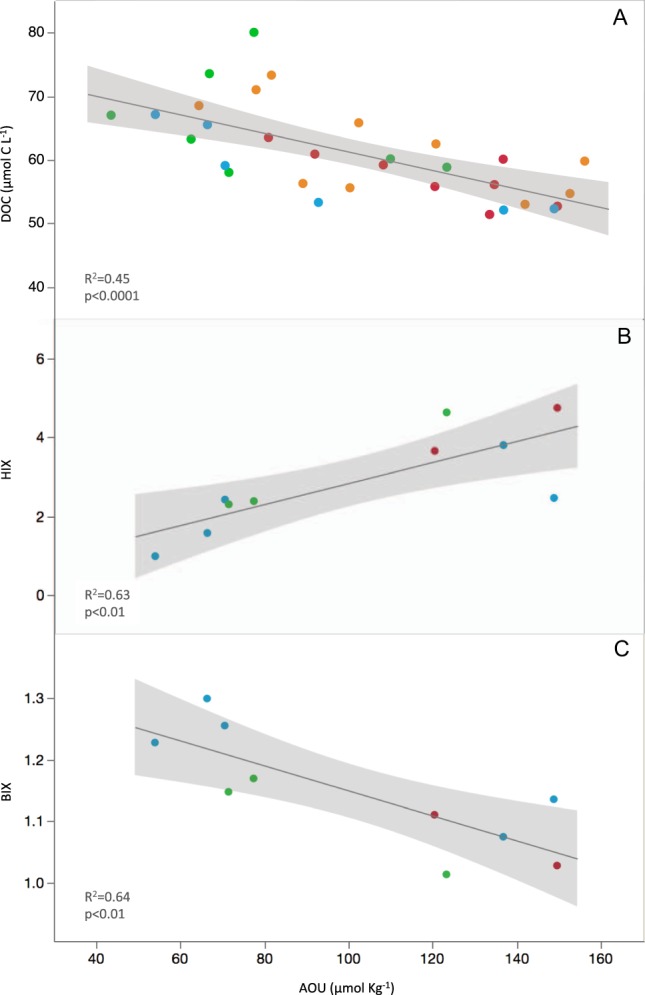
DOC, HIX and BIX correlations with AOU at isopycnal 4. The relationship of DOC (µmol C L^−1^) (**A**), HIX (**B**) and BIX (**C**) with AOU (µmol Kg^−1^) within the isopycnal 4 (σ_θ_ = 27.8–28.4 Kg m^−3^). Seasons are indicated in different colors: Winter (blue), Spring (green), Summer (red) and Fall (orange). Black line represents the fit that follows equations (**A**) DOC = −0.15 (±0.03) AOU + 75.87 (±3.24), r ^2^ = 0.45, p < 0.0001, (**B**) HIX = 0.03 ( ± 0.01) AOU + 0.17 (±0.79), r^2^ = 0.63, p < 0.01 and (**C**) BIX = −0.002 (±0.001) AOU + 1.35 (±0.06), r^2^ = 0.64, p < 0.01. The shaded area represents the confidence intervals for every fit line.

**Figure 6:**
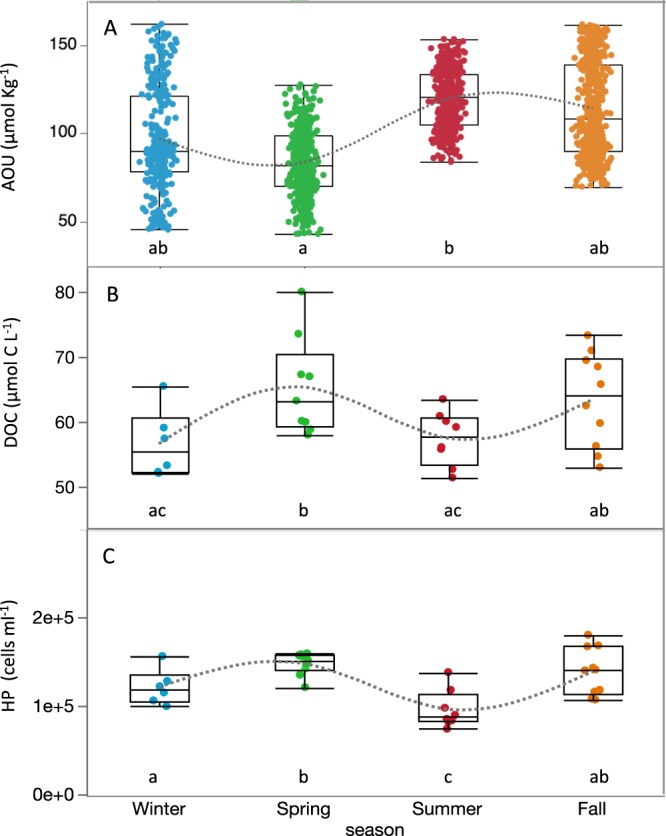
Seasonality of AOU, DOC and HP at isopycnal 4. Seasonal variability of AOU (µmol Kg^−1^) (**A**), DOC (µmol C L^−1^) (**B**) and HP abundance (cells mL^−1^) (**C**) within the isopycnal layer 4 (σ_θ_ = 27.8–28.4 Kg m^−3^). Seasons are indicated in different colors: Winter (blue), Spring (green), Summer (red) and Fall (orange).

In the isopycnal 4 (σ_θ_ = 27.8–28.4 Kg m^−3^), mostly between 100 and 200 m, we found DOC concentration to significantly decline with increasing AOU (DOC = −0.15 (±0.03) AOU + 75.87 (±3.24), r = −0.67, p < 0.0001, n = 31, Fig. 5A, Supplementary Table 2), indicating a substantial contribution of DOC remineralization to changes in AOU within that isopycnal. To evaluate the fraction of oxygen consumption related to DOC, AOU was converted to carbon equivalents, by using the molar ratio ΔC:ΔAOU = −0.72 according to Anderson (1995)^51^. A slope of −0.72 would be expected if all oxygen consumption was related to the remineralisation of DOC. Thus, our slope (−0.15) indicates that 21% of changes in AOU were driven by DOC respiration at this density horizon. Significant increase in HIX (r = 0.79, p < 0.01, n = 10) and decrease in BIX (r = −0.80, p < 0.01, n = 10) with increasing AOU were also concurrently observed (Fig. 5B,C, Supplementary Table 2), indicating that humic-like material was being produced during remineralization of semi-labile DOC, while freshly produced biological material was being consumed. AOU was also significantly correlated with DIN (r = 0.86, p < 0.0001, n = 25) and DIP (r = 0.66, p < 0.001, n = 25) (Supplementary Fig. 3A,B, Supplementary Table 2). AOU presented seasonal variability within this layer (Fig. 6A), with significantly lower mean values in spring (84 ± 19 µmol Kg^−1^) than summer (120 ± 17 µmol Kg^−1^) (p < 0.05, posthoc Fisher LSD test). DOC concentration and HP abundance displayed a quasi-identical and inverse seasonal pattern to AOU, both showing significantly higher mean values in spring (65.3 ± 7.5 µmol C L^−1^) than winter and summer (ranging from 51.4 to 65.5 µmol C L^−1^) (p < 0.05, posthoc Fisher LSD test) (Fig. 6B,C).

Isopycnal 5 (σ_θ_ = 28.4–28.8 Kg m^−3^), from around 200 m all the way down to the seafloor (i.e. coincident with most of the mesopelagic layer), showed no correlation between DOC and AOU, but here HIX also increased significantly with increasing AOU levels (r = 0.48, p < 0.01, n = 32) (Supplementary Fig. 4, Supplementary Table 2) at a higher rate than at isopycnal 4 (slope = 0.08 v. 0.03). AOU correlations with DIN and DIP were still significant but weaker than at isopycnal 4 (r = 0.27 and 0.24, p < 0.05, n = 74, respectively, Supplementary Table 2).

### Relationships between DOC and heterotrophic prokaryotes

With all data pooled, HP abundance was positively correlated with DOC (R = 0.76, p < 0.001, n = 246). The correlation was weaker but still significant in the epipelagic layer (R = 0.44, p < 0.001, n = 130) but it was lost in mesopelagic waters. If we zoom into the previously defined isopycnal layers, we observe that HP biomass was significantly and positively correlated with DOC (R = 0.50, p < 0.005, n = 34) only at the isopycnal 4 where DOC remineralization significantly contributed to changes in AOU as Fig. 6B,C already suggested.

## Discussion

The seasonal hydrography observed (Fig. 1) revealed that the sampling station was affected by the global thermohaline circulation of the Red Sea, in turn controlled by the seasonal patterns of the monsoon winds^52^. The inflow of Indian ocean surface waters through the Gulf of Aden due to wintertime northward winds was exhibited in surface waters (above 100 m) with low salinity and temperature values in that season (Fig. 1 and Supplementary Fig. 1). In contrast, surface waters during summer and fall displayed higher salinity and temperature due to southward winds, which create an inflow from the northern Red Sea where high evaporation rates generate hot and highly saline surface waters^53^ (Supplementary Fig. 1). High salinity Red Sea waters travel further south and enter the northwest Indian Ocean through a shallow sill located at the Strait of Bab el Mandeb, sinking to intermediate waters to more than 1000 m as far south as 10°S^54^. This water mass, additionally fed by the outflow of the also exceptionally saline Persian Gulf is known as the Red-Sea-Persian Gulf Intermediate Water mass (RSPGIW)^55^ and seasonally contributes to highly saline deep waters in the Indian Ocean with sigma theta values between 27 and 28 Kg m^−3^, very similar to the values observed at isopycnal 3 (27.1–27.8 Kg m^−3^).

The seasonal thermocline observed over the period studied (2014–2017) was shallower (between 20 and 100 m) than documented three decades ago for the central Red Sea (between 150 and 200 m at 20–25°N^56^), likely as a consequence of the recent fast warming^15^. Our data also likely captured the influence of 2015 El Niño event (2015 displayed maximum temperatures 1.7 °C higher than those observed during 2016), when maximum temperature anomalies had dramatic consequences for the health of coastal ecosystems of the Red Sea^15^. This supports the mechanism proposed by Raitsos *et al*.^57^ suggesting that the monsoon’s seasonality and the global climate Multivariate El Niño-Southern Oscillation (ENSO) are influencing the Red Sea marine ecosystem functioning. We can infer from the hydrological features observed that our station was not isolated from the seasonal or inter-annual processes affecting the Red Sea as a whole.

As usual for most tropical environments unaffected by upwelling, close to zero or below detection limit values of DIN and DIP at the surface layer were observed^58,59^ and increased with depth. Values recorded within the mesopelagic layer were similar to previous measurements in the Red Sea^60^, and as much as one order of magnitude higher than the Mediterranean Sea^31^ suggesting a higher microbial remineralization of nitrogen and phosphorus in the mesopelagic waters of the Red Sea. The DIN:DIP rations in this layer (23 ± 7), higher than the Redfield ratio and suggesting phosphorus limitation, were similar to previous measurements in the mesopelagic Red Sea^60^ and comparable to those in the ultraoligotrophic Eastern Mediterranean Sea subsurface waters^31^. Epipelagic waters, on the contrary, generally showed lower DIN:DIP ratios (∼11), indicating N limitation for most of the year except in summer (DIN:DIP up to 19), with implications for the distribution and dynamics of nitrogen fixing diazotrophs (*Trichodesmium*), commonly abundant in N limiting waters of the Red Sea^60^.

The seasonality of HP abundance in the epipelagic displayed a slightly different pattern than DOC, with an increase in abundance from winter to spring followed by a decrease in summer (Fig. 2C), contrary to seasonal patterns reported in the Sargasso Sea^35^ but similar to those in the Pacific Ocean for the year 1992 surveyed in Campbell *et al*.^61^. The lower summer HP abundance might indeed have allowed the accumulation of DOC observed in this season (Fig. 2E). Summer high DIN:DIP ratios suggest that HP growth might have been limited by inorganic phosphorus or that there was a strong bacteria-phytoplankton competition for this nutrient, thus decreasing DOC consumption ultimately leading to its accumulation, as previously hypothesized^62^. Similar results have been reported for Mediterranean surface waters where low bacterial growth efficiencies were linked to DOC accumulation during summer stratification^63^. The low BIX and FI values (means of 1.1 and 1.4, respectively) observed in the epipelagic in summer could have different explanations. According to the traditional interpretation^43^, low values of both indices may suggest a smaller proportion of freshly produced, most likely labile phytoplanktonic DOM accompanied by a stronger terrestrial signature, which we could attribute to the influence of mangrove-derived material from nearby coastal waters^64^. However, this interpretation should be taken with caution since recent research has identified other/novel microbial processes affecting the FDOM signature in the oligotrophic ocean. Picocyanobacteria (*Synechococcus* and *Prochlorococcus*) abundant in the Red Sea can release FDOM with a humic-like appearance^44^, while *Trichodesmium*, also important in these tropical N-limited waters, can as well produce chromophoric DOM in the open ocean^65^. Higher predation on HP or mortality caused by viruses could also be partly responsible for the decrease in HP abundance and subsequent DOC accumulation^62^.

Our DOC epipelagic observations did not differ from oligotrophic areas elsewhere, with similar values and seasonal accumulation when the water column was more stratified^35,63,66,67^. The slope of the DOC change with depth along the thermocline, smaller in winter than in summer (Supplementary Fig. 2A), revealed the downward export of accumulated semi-labile DOC to subsurface waters when the mix layer deepens during winter. The semi-labile nature of subducted DOC was confirmed by the significant contribution to oxygen consumption (21%) at the isopycnal 4 (density horizon σ_θ_ = 27.8–28.4 Kg m^−3^) (Fig. 5A). We hypothesize that processes responsible for the remaining 79% are associated to respiration of sinking POC due to particle-associated bacteria, zooplankton and other heterotrophic marine organisms^68^. DOC contribution to oxygen consumption is similar to those reported for the Mediterranean^31^ and Sargasso Sea^5^ but smaller than that documented for the vast subtropical gyres (>50%^13,30,34^). The correlation of DOC concentration with HP abundance and their similar seasonal pattern suggests that temporal changes in available DOC were driving changes in HP abundance evidencing a tight bottom-up control of HP by substrate availability within this layer. Optical indices indicate that while DOC was being consumed, the remains of it were enriched in humic-like substances (HIX increased) and depleted in autotrophic freshly produced DOC (BIX decreased) (Fig. 5B,C). This is in accordance with reported increase of humic-like and decrease of protein-like fluorescent substances during microbial remineralization for the global ocean^26^. The consumption of molecules with high BIX index further evidence the semi-labile nature of subducted DOC. Moreover, the significant increase in DIN and DIP with AOU within this layer indicated that respiration of DOC not only contributed to the remineralization of organic carbon but also to that of dissolved organic nitrogen and phosphorus thus yielding an increase in DIN and DIP values during oxygen consumption (Supplementary Fig. 3).

In the mesopelagic zone (>200 m), below the above-mentioned isopycnal layer, AOU values reached its maximum at around 300 m and remained relatively constant (190 ± 5 µmol Kg^−1^) all the way down to the sea floor (ca. 700 m). However, DOC concentrations below 200 m displayed values varying between 42 and 58 µmol C L^−1^ (Figs 2B and 3B), revealing a higher than expected variability (>15 µmol C L^−1^) for what is commonly regarded as an homogenous mesopelagic layer (with DOC variability ∼5 µmol C L^−1^ ^25^). This observation, together with the fact that no significant relationship between DOC and AOU was found, neither with HP that remained relatively constant in the mesopelagic (Fig. 2A), made us recall other important processes occurring within it that may affect carbon fluxes. A large portion of deep water DOC can have a fast turnover time supported by the dissolution of sinking POC, with consequences for annual carbon fluxes throughout the water column^69^. The mesopelagic Red Sea is also home to vertically migrating fish, mostly dominated by skinnycheek lanternfish (*Benthosema pterotum*), which feed year-round at the surface at night and rest between 400 and 600 m during daytime forming a conspicuous deep scattering layer (DSL)^70,71^. Recent reports of high bacterial growth efficiencies measured at the DSL of the same study site seemingly sustained by DOC supplied through fish migration^10^, suggest the existence of complex and still poorly explored interactions between daily fluxes of DOC and the microbial communities thriving in these layers. The significant increase in humification index with AOU (Supplementary Fig. 4) and HIX increased values between 400 and 600 m (Fig. 4B) could be due to different processes: i) microbial production of highly humified DOC in the mesopelagic supporting previous studies that suggest a diverse and dynamic community of mesopelagic heterotrophic prokaryotes within the DSL^11^; ii) chromophoric DOM release from vertically migrating fish through excretion, fecal pellet dissolution and/or mucus production, all of them recognized mechanisms of DOM supply from migrating animals^65,72^, likely fuelling bacterial activity in the mesopelagic; iii) picocyanobacteria derived-DOM efficiently transported to the deep sea^73^ with humic-like FDOM properties^44^. Calleja and co-workers (2018)^10^ also reported high relative abundances of *Candidatus “Nitrosopumilus maritimus”* in these waters (40–50% of the total prokaryote abundance), an archaeon described to be responsible for most of ammonium oxidation and inorganic carbon fixation in the deep ocean^74^. We hypothesize that the DOC variability observed in the mesopelagic could be explained by the mixotrophic metabolic capabilities of the abundant marine archaeal community^74–76^ most probably fuelled by the supply of ammonia from the migrating fish^77^, further contributing to reduced oxygen levels (revealed by the very high AOU values >300 m) and enhanced DIN values in the mesopelagic.

This work is the first seasonal evaluation of DOC in the Red Sea, one of the least quantified and understood bioreactive pools of carbon. Our results reveal seasonal semi-labile DOC accumulation in the surface waters, which subduction and remineralization mostly occurred within a specific density horizon located between 100 and 200 m depth. DOC respiration contributed to 21% of oxygen consumption making a small but significant contribution towards the maintenance of respiratory processes in the Red Sea, particularly in the low epipelagic/upper mesopelagic boundary layer. In the deeper mesopelagic layer processes other than DOC respiration seemed to be dominating changes in dissolved oxygen consumption. We hypothesize that the addition of short-lived labile substrates most likely excreted by daily migrating fish, may stimulate microbial metabolism and enzyme production of both heterotrophic prokaryotes and mixotrophic archaea co-habiting these waters therefore facilitating DOC degradation/transformation and increasing humification index. Remineralized DOC products in subsurface waters may return slowly to surface waters by ventilation processes (i.e., decades to centuries) and are important to consider when evaluating deep sea carbon sequestration^16^. Microbially mediated DOC transformation processes may be intensified by the high temperatures that the Red Sea experiences. Whether this could be the case of other oligotrophic oceanic basins threaten by increasing warming is yet to be explored.

## Supplementary information

Supplementary Information

## Data Availability

All data needed to evaluate the conclusions in the paper are present in the paper and/or the Supplementary Information.
