# Supplementary material for: Dissolved organic carbon contribution to oxygen respiration in the central Red Sea
Source: Sci Rep. 2019 Mar 18;9:4690. doi: 10.1038/s41598-019-40753-w (PMC6423344; doi:10.1038/s41598-019-40753-w)
Supplement: Supplementary file 1 — Supplementary Information [file 41598_2019_40753_MOESM1_ESM.pdf]

# Supplementary Information

# Dissolved organic carbon contribution to oxygen respiration in the central Red Sea

**Maria Ll. Calleja[1*], Najwa Al-Otaibi[1] and Xosé Anxelu G. Morán[1]**

Remove the super-index “1” and “*”.

Figure 1. T-S (Potential Temperature-Salinity) diagram of all vertical profiles sampled within the two-year sampling period showing that the different water masses presents followed a seasonal pattern. The four natural seasons are marked with different colors: Winter (blue), Spring (green), Summer (red) and Fall (orange).

Figure 2. Seasonal variability of the slope of DOC vs depth ($\Delta$DOC/$\Delta$z, µmol C $L^{-1}$ $m^{-1}$) in the thermocline (A), and seasonal variability of the central depth from which the slope in A was calculated (B). Seasons are indicated in different colors: Winter (blue), Spring (green), Summer (red) and Fall (orange).

Figure 3. Relationships between AOU and dissolved inorganic nitrogen (DIN, A) and phosphorus (DIP, B) at the isopycnal layer $\sigma\theta$ = 27.8-28.4 Kg $m^{-3}$. Seasons are indicated in different colors: Winter (blue), Spring (green), Summer (red) and Fall (orange).

Figure 4. Relationships between HIX and AOU (µmol $Kg^{-1}$) at the deeper isopycnal layer $\sigma\theta$ = 28.4-28.8 Kg $m^{-3}$. Seasons are indicated in different colors: Winter (blue), Spring (green), Summer (red) and Fall (orange).

Table 1. Seasonal pattern, ANOVA significance (p-value) and posthoc Fisher LSD test results for the epipelagic and mesopelagic layers of Sigma-Theta ($\sigma_{\theta,}$ Kg $m^{-3}$), vivo Chla fluorescence (Chla Fluor, R.U.), AOU (µmol $Kg^{-1}$), DIP:DIN molar ratio, heterotrophic prokaryotes abundance (HP, cells $ml^{-1}$), DOC (µmol C $L^{-1}$) and its corresponding fluorescent indices (FI, BIX and HIX).

Replace “layer” by “layers”.

Table 2. Pearson correlations results (r, p-value, slope, intercept, n) from the relationship between DOC, its corresponding fluorescent indices (HIX, BIX) and dissolved inorganic nutrients (DIN and DIP) with AOU, within each of the isopycnal layer. Isopycnals 2 and 3 reported together because the number of observations within the thin isopycnal 3 were below 5. Significant correlations are marked in bold.

Add “are” between “3” and “reported”.

Replace “were” by “was”.

*note1* : * indicates a p-value < 0.05, ** indicates a p-value < 0.01, *** indicates a p-value < 0.001, **** indicates a p-value < 0.0001

*note3* : to be able to compare it with previously published results, when examining the relationship between AOU and dissolved inorganic nutrients AOU is regarded as the dependent variable and DIN and DIP as the independent variables.

Fig. 1

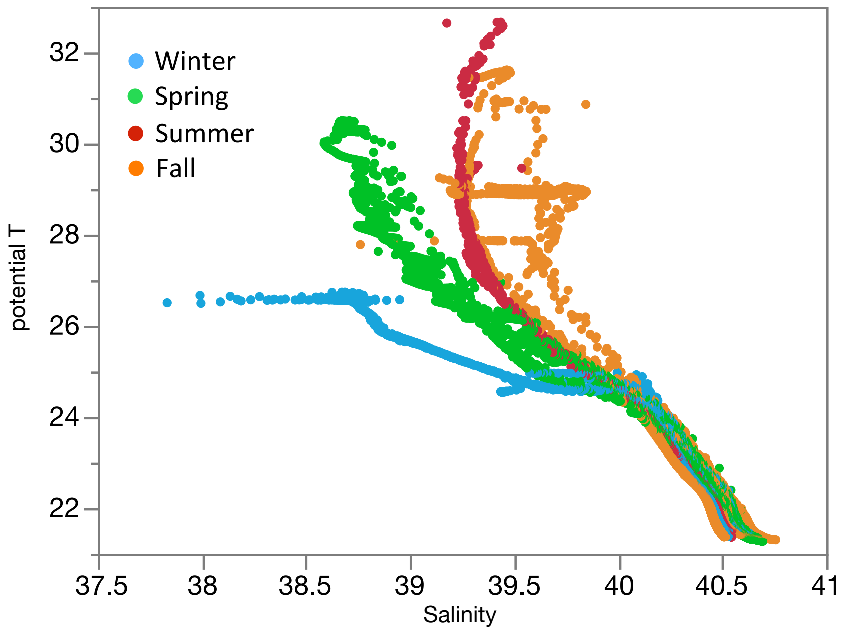
Winter
Spring
Summer
Fall
potential T
Salinity
32
30
28
26
24
22
37.5
38
38.5
39
39.5
40
40.5
41

Fig. 2

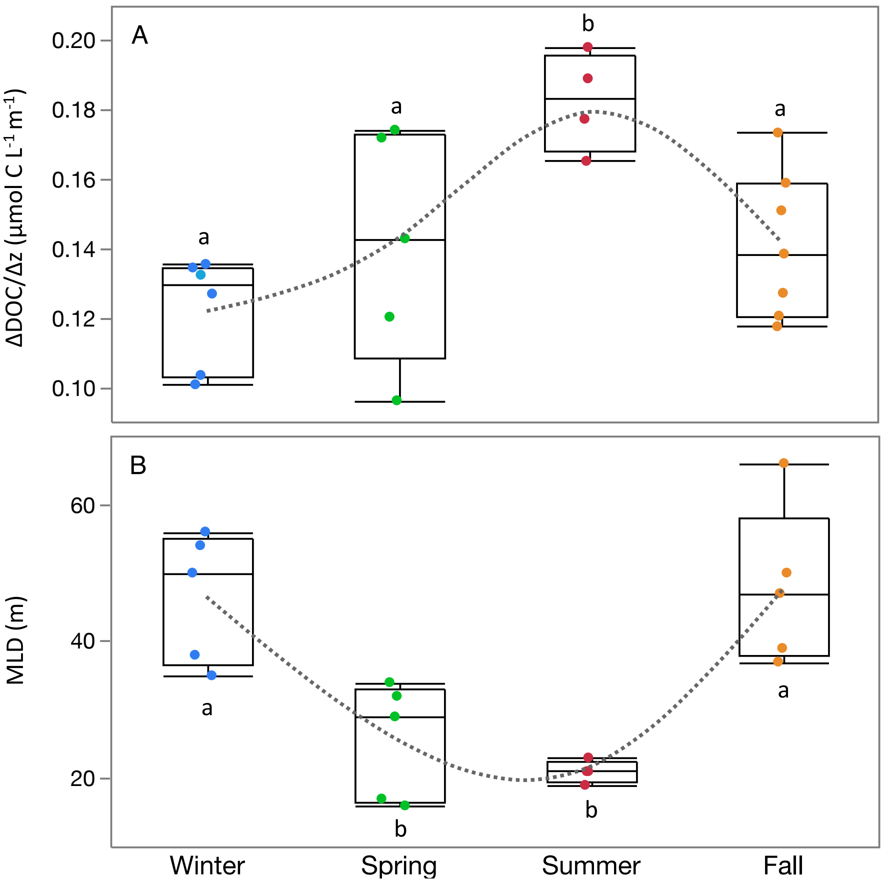
A
B
ΔDOC/Δz (μmol C L-1 m-1)
MLD (m)
0.20
0.18
0.16
0.14
0.12
0.10
60
40
20
a
b
Winter
Spring
Summer
Fall

Fig. 3

Fig. 3 is missing the “A” and “B” legend. I have added it manually to this file

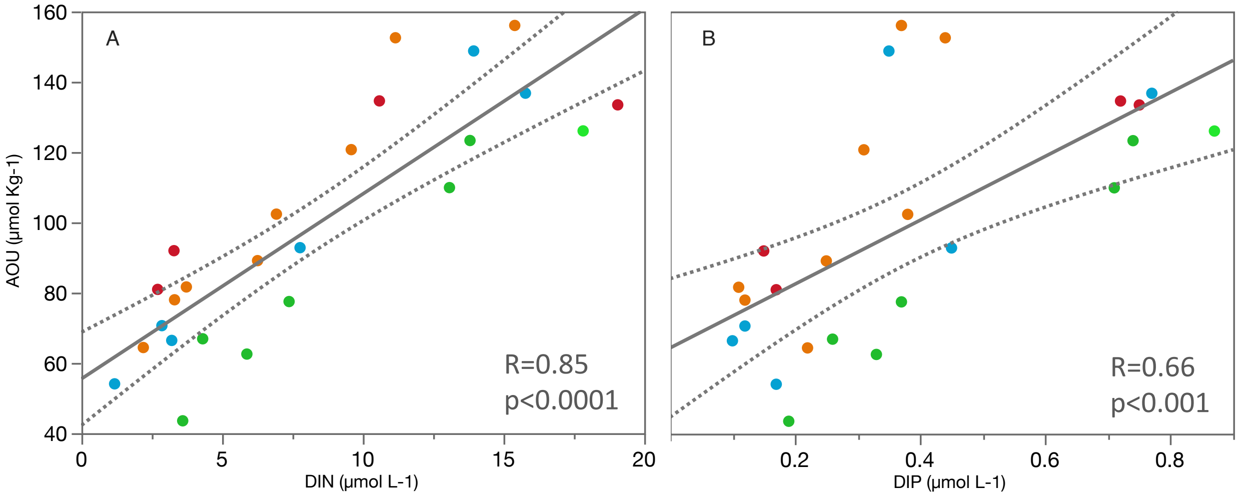


To be able to compare it with previously published results, when examining the relationship between AOU and dissolved inorganic nutrients AOU is regarded as the dependent variable and DIN and DIP as the independent variables.

Pooling all profiles together, AOU significantly increased with increasing DIN ($R=0.93$, $p<0.0001$, slope=6.7) and DIP ($R=0.88$, $p<0.001$, slope=130), displaying oxidative ratios (slopes) of the same magnitude but smaller than those previously reported for the Red Sea (11 for $NO_3^-$ and 230 for $PO_4^{3-}$)[1].

Fig. 4

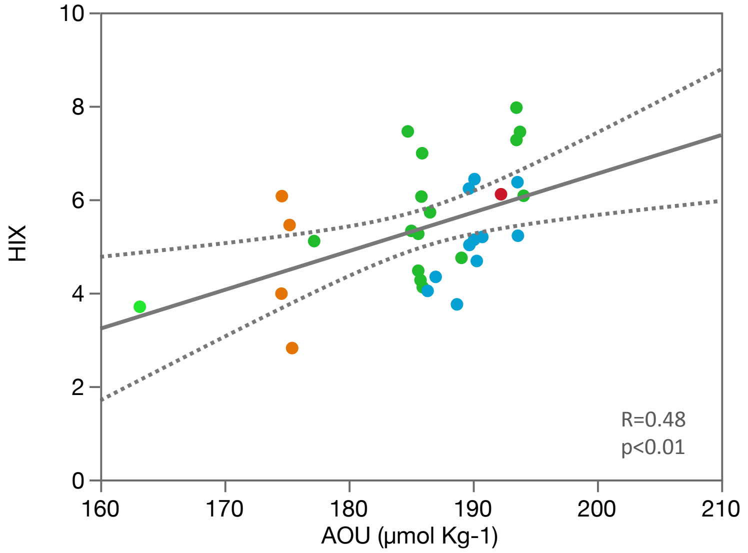

Table 1

| | epipelagic | | | | | mesopelagic | | | | |
|---|---|---|---|---|---|---|---|---|---|---|
| | p-value | W | Sp | S | F | p-value | W | Sp | S | F |
| Sigma-T | *** | a | a | b | c | *** | a | b | c | d |
| Chla Fluor | *** | a | b | c | d | NA | | | | |
| AOU | *** | a | b | c | d | *** | a | ab | c | b |
| DIN:DIP | ** | a | a | b | a | ns | a | a | a | a |
| HP | ns | a | a | a | a | *** | a | a | b | a |
| DOC | ** | a | b | b | b | *** | a | b | b | b |
| FI | * | a | a | b | a | ns | a | a | a | a |
| BIX | ns | a | a | a | a | ns | a | a | a | a |
| HIX | ns | a | a | a | a | ns | a | a | a | a |

Table 2

| isopycnal | DOC (µmol C L-1) | | | | | HIX | | | | | BIX | | | | | DIN (µmol N L-1) | | | | | DIP (µmol P L-1) | | | | |
|---|---|---|---|---|---|---|---|---|---|---|---|---|---|---|---|---|---|---|---|---|---|---|---|---|---|
| | r | p-value | slope | intercept | n | r | p-value | slope | intercept | n | r | p-value | slope | intercept | n | r | p-value | slope | intercept | n | r | p-value | slope | intercept | n |
| 1 | 0.32 | ns | 0.06 | 70.2 | 69 | -0.14 | ns | -0.002 | 1.667 | 23 | -0.22 | ns | -4E-04 | 1.176 | 23 | **0.61** | *** | **27.11** | **40.2** | **67** | **-0.32** | * | **-109.1** | **61.6** | **67** |
| 2&3 | -0.31 | ns | -0.09 | 71.2 | 15 | 0.02 | ns | 0.000 | 1.974 | 7 | -0.68 | ns | -0.001 | 1.262 | 7 | -0.14 | ns | -3.554 | 44.0 | 15 | -0.19 | ns | -54.7 | 53.14 | 15 |
| 4 | **-0.67** | **** | **-0.15** | **75.9** | **31** | **0.79** | ** | **0.027** | **0.172** | **10** | **-0.80** | ** | **-0.002** | **1.354** | **10** | **0.86** | **** | **5.26** | **55.5** | **25** | **0.66** | *** | **90.86** | **64.3** | **25** |
| 5 | 0.20 | ns | 0.12 | 27.6 | 83 | **0.48** | ** | **0.083** | **-10.03** | **32** | **-0.36** | ** | **-0.002** | **1.238** | **32** | **0.27** | * | **0.9446** | **167.1** | **74** | **0.24** | * | **11.8** | **174.7** | **74** |

## References

1. Naqvi, S. W. A., Hansen, H. P. & Kureishy, T. W. Nutrient uptake and regeneration ratios in the Red Sea with reference to the nutrient budgets. *Oceanol. Acta* **9,** 271–275 (1986).
